# Healthy city evaluation based on factor analysis—Taking cities in the Guangxi Zhuang Autonomous Region as an example

**DOI:** 10.1371/journal.pone.0306344

**Published:** 2024-07-12

**Authors:** Hui Huang, Shuxin Huang, Shaoyao He, Yong Lu, Shuguang Deng

**Affiliations:** 1 Guangxi Natural Resources Vocational and Technical College, Nanning, China; 2 School of Architecture, Hunan University, Changsha, China; 3 Guilin University of Technology at Nanning, Chongzuo, China; 4 School of Geography and Planning, Nanning Normal University, Nanning, China; Guangxi Normal University, CHINA

## Abstract

As urbanization speeds up, the concept of healthy cities is receiving more focus. This article compares Chongzuo and Nanning in Guangxi with Beijing to assess the development gaps in cities in Guangxi. An indicator system for healthy cities was designed from six dimensions—healthy economy, healthy population, healthy healthcare, healthy environment, healthy facilities, and healthy transportation—and 26 secondary indicators, which were selected from 2005 to 2022, and an improved factor analysis was used to synthesize a healthy city index (HCI). The number of factors was determined by combining characteristic roots and the variance contribution rate, and the HCI was weighted using the entropy-weighted Topsis method. A comprehensive evaluation of the urban health status of these cities was conducted. The results showed that extracting six common factors had the greatest effect, with a cumulative variance contribution rate of 93.83%. Chongzuo city scored higher in the field of healthcare. The healthy environment score of Nanning was relatively high, which may be related to continuous increases in green measures. In terms of the healthy economy dimension, Beijing was far ahead. However, in recent years, the healthy economy level in Chongzuo has increased, and the GDP growth rate has ranked among the highest in Guangxi. In addition, the growth rate of healthy facilities in Nanning was relatively fast and has been greater than that in Chongzuo in recent years, which indicates that the Nanning Municipal Government believes urban construction and municipal supporting facilities are highly important. In terms of healthy transportation, Chongzuo and Nanning scored higher than Beijing. This may be because the transportation in these two cities is convenient and the traffic density is more balanced than that in Beijing, thereby reducing traffic congestion. Chongzuo had the highest score for a healthy population, and a steadily growing population provides the city with stable human resources, which helps promote urban economic and social development. Finally, relevant policy recommendations were put forwards to enhance the health level of the cities.

## 1. Introduction

As urbanization causes issues such as worsening environmental pollution, frequent health incidents, limited public services, and severe traffic emissions, the construction of healthy cities has become a global strategic initiative, garnering consensus among nations [1]. The definition of a healthy city by the World Health Organization (WHO) in 1994 is: "A healthy city should be a city that continuously develops and develops its natural and social environment, and continuously expands its social resources so that people can support each other in enjoying life and fully realizing their potential.". With the development of society, Professor Fu Hua and others from the School of Public Health at Fudan University in Shanghai have further proposed a more easily understandable definition: "A healthy city refers to a development whole that centers on human health in all aspects of urban planning, construction, and management, ensuring the healthy life and work of the general public, and becoming a necessary healthy population, healthy environment, and healthy society for the development of human society". Many countries and regions have explored and implemented healthy city principles [2]. For example, Europe established a healthy city network [3], the United States and other countries established a healthy city and community alliance, and South Korea and other countries launched the Healthy City and Community Construction Project [4]. China issued the "Healthy China 2030" Planning Outline, which proposes constructing healthy cities as an important means of promoting the construction and development of a healthy China and selects 38 cities to be part of 2016 Healthy China pilot program for urban construction [5]. For China, promoting healthy cities is crucial for implementing the Healthy China strategy and will significantly influence its future sustainable development.

However, due to varying development endowments such as location, history, culture, economy, resources, and environment among Chinese cities [6], the level of healthy city construction and the effectiveness of the pilot program’s policies vary and are inconsistent [7]. The literature has mostly focused on the development of first-tier cities or new first-tier cities, and little attention has been devoted to non-first-tier cities, where problems such as environmental pollution, traffic congestion, and urban poverty are common [8]. In addition, among the non-first-tier cities, the Guangxi Zhuang Autonomous Region has a unique geographical location and rich ethnic culture, which gives it unique research value. However, existing research has not paid enough attention to Guangxi cities. As the capital and political, economic, and cultural hub of Guangxi, Nanning faces urban and environmental challenges. Studying these issues can provide crucial insights for the sustainable development of China’s second- and third-tier cities [9]. In addition, Nanning city, as the transportation hub of Guangxi, is also highly important for studying urban transportation issues [10]. At the National Healthy Cities, Healthy Villages and Towns Construction Symposium and Healthy City Pilot Launching Meeting held in Hangzhou in 2023, Nanning was identified as one of the first 38 healthy city pilot cities in the country. However, it was the only city in Guangxi that was selected. Studying Nanning city helps to evaluate the effectiveness and development of healthy cities in China [11]. Second, Chongzuo city, another prefecture-level city in Guangxi, boasts natural resources and cultural heritage, such as the Detian Waterfall and Youyi Pass. As a border trade hub, it is vital for studying border area economic development. During the pandemic, Chongzuo’s strong epidemic control measures were noteworthy, and the city had no new local cases for a prolonged period.

Therefore, this paper will focus on measuring the health levels of Nanning and Chongzuo cities in the Guangxi Zhuang Autonomous Region to fill the gaps and deficiencies in the research in this area. In addition, by constructing a healthy city index (HCI) and comprehensively evaluating the level of healthy city construction in the Guangxi Zhuang Autonomous Region, this study can provide a scientific basis for promoting the sustainable development of healthy cities and achieving a higher level of national health. This is relevant not only to Guangxi’s development but also to the pursuit of national health. This study uses Beijing as a benchmark city for comparison based on indicator data from 2005 to 2022 and uses Nanning city and Chongzuo city in the Guangxi Zhuang Autonomous Region as the research objects to measure the level of healthy city construction in each city, synthesize a healthy city index (HCI), and compare the development differences and longitudinal development prospects of the cities. By constructing an urban health evaluation system and using factor analysis for a comprehensive evaluation, we hope to provide useful insights for the comprehensive, balanced, and sustainable development of healthy cities in the Guangxi Zhuang Autonomous Region.

## 2. Literature review

Healthy cities have received increasing attention [12]. A review of relevant domestic and foreign literature shows that existing research has mainly focused on the conceptualization of healthy cities [13], development practices [14], measurement and evaluation [15, 16], impact effects [17], and enhancement paths [18].

Some of these studies have focused on the measurement and evaluation of healthy urban development and construction. Li, Fang, and Zeng constructed a healthy city evaluation index system considering four aspects—the environment, society, services, and behaviour—and evaluated and analysed the development of Chongqing [19]. Ding et al. constructed an evaluation system for healthy city construction in Ningbo from the aspects of health culture, health status, and healthy society and conducted an evaluation of the coupling and coordination relationship between healthy city construction and urbanization development in Ningbo [20]. De Leeuw E proposed a method for evaluating complex urban projects, constructed the World Health Organization European Healthy Cities Network, reviewed the basic principles and parameters of the project, emphasized that healthy city research should proceed from the perspective of multilevel, reciprocal paths, and conducted research on the impacts and outcomes of healthy cities. In addition, the study reflected on the evaluation of the fourth phase (2003–2008) and explained the response rates of various methods [21]. Wang et al. constructed a healthy city evaluation system by reviewing the connotations of healthy cities and used a combined model based on the analytic hierarchy process and entropy weighting to evaluate the performance of healthy city construction in 284 Chinese cities. The results showed that the average distribution level of China’s healthy city construction performance showed an "N-shaped" trend, fluctuating upward from 2009 to 2019, but with an overall relatively low level [5].

In addition, other scholars have studied solutions for healthy urban development. For example, Krecl et al. systematically analysed the impact of several traffic management strategies (bus lanes, bike lanes, traffic calming zones, traffic lights, and clean vehicle technologies) on urban air pollution by planning new bike lanes and adjusting traffic signals [22]. Fan and Zheng found that dockless shared bicycles help achieve greener and healthier cities by supplementing subway travel and alleviating road congestion [23]. Oviedo and Sabogal-Cardona reported that the potential to reduce car use and increase bicycle travel could lead to greater social gains in sustainable urban health [24]. Barton and Grant selected data from European cities in the fourth phase of healthy city planning and used evaluation methods to evaluate European cities. The results showed that health conditions are an important factor affecting urban health. Three levels of health and planning integration were also identified, providing standards for assessing progress [25]. Balaban O and Oliveira studied the impact of population and economic activity in urban areas on the link between cities, health, and the environment and found that the concept of sustainable (green) buildings can generate multiple benefits. They selected data from case study buildings, used performance evaluation methods to evaluate urban buildings, and found that green buildings can significantly reduce energy use intensity and carbon dioxide emission intensity, reduce costs, and improve the health status of building users [26]. Kegler and Twiss selected more than 40 cities participating in the California Healthy Cities Project as research objects and used a comprehensive evaluation framework to conceptualize changes in healthy cities at five levels: individual, citizen participation, organization, interorganizational, and community. They worked with healthy city actors to develop the framework, which attempted to integrate concepts such as community capacity, social ecology, and urban planning to evaluate ideas and practices in community projects [27]. Sharifi A noted that healthy city assessment tools should leverage advances in smart solutions and big data analytics to develop better strategies to meet these healthy city developments [28]. Nieuwenhuijsen selected research data on the relationship between urban and transportation planning and public health and used a meta-review method to evaluate the impact of urban and transportation planning on public health. The results showed that better transportation planning can improve public health as well as measures such as urban greening. These measures can also make cities more sustainable and liveable, resulting in multiple benefits [29]. Kingman et al. studied the impact of the urban heat island effect (UHI) on the health of European urban residents and assessed the role of increasing urban forest coverage in reducing temperatures and preventing premature death [30]. In addition, Sun H et al., based on panel data of 201 cities from 2011 to 2020, studied the application of the spatial Durbin model and threshold regression model and identified heterogeneous agglomeration in the eastern and western parts of China [31]. Finally, some of the literature has focused on listed companies [32] or foreign regions [33].

In summary, although the field of healthy city research has achieved a series of valuable results, existing research has mainly concentrated on first-tier cities, and there have been relatively few studies on cities in non-first-tier and border ethnic areas. Further, the research factors or perspectives evaluated in the previous research have been relatively simple, making it difficult to perform a more systematic comprehensive evaluation. In addition, comparative analyses of non-first-tier and first-tier developed cities are lacking. In particular, there are very few studies on healthy cities in Guangxi in the literature. As the bridge and link of the China–ASEAN community, which includes countries that have a shared future, Guangxi has unique location advantages and geographical characteristics. Evaluating the health of cities in Guangxi is of great practical significance for promoting high-quality development, social security, and stability in border ethnic areas, thus promoting social modernization governance. Therefore, researching cities in the Guangxi Zhuang Autonomous Region can help local governments better understand and grasp the laws related to urban development and construction in ethnic minority areas to improve the overall health of the city. Moreover, by comparing these cities with first-tier cities such as Beijing, it is easy to discover the differences and deficiencies of the cities in the Guangxi Zhuang Autonomous Region in terms of the economy, culture, society, and other aspects. This study provides a more comprehensive and scientific reference for urban construction when studying urban development in the future. To fill this research gap, this paper takes Nanning city and Chongzuo city in the Guangxi Zhuang Autonomous Region as examples and uses the factor analysis method to systematically measure and evaluate the healthy city construction levels of these two cities. This study provides a decision-making reference for improving the health level of Chinese cities and for improving policy regulation.

The contributions of this paper are summarized in the following points:

1. Based on the characteristics of cities in Guangxi, this paper constructed a relatively systematic urban health evaluation system covering multiple aspects, including the economy, population, medical care, environment, facilities, and transportation, making urban health evaluation more comprehensive and scientific. A quantitative measurement analysis was conducted on two cities in the Guangxi Zhuang Autonomous Region, Nanning and Chongzuo, further deepening the research on healthy cities.
2. An improved factor analysis was used for the comprehensive evaluation, which improves the subjectivity of the traditional factor analysis method in determining the number of factors and synthesizing a comprehensive index. This improved factor analysis can objectively measure a city’s health level and avoid the influence of subjective factors on the evaluation results.
3. By focusing on cities in border ethnic areas such as Nanning and Chongzuo in Guangxi as examples and taking Beijing as the benchmark city for comparison, the shortcomings of the healthy development of cities in Guangxi can be highlighted, and the results showed that these two cities in Guangxi are experiencing economic development and exercising environmental protection and have social resources. Shortcomings in other aspects provide a basis for formulating targeted development strategies.

## 3. Research methods

Currently, commonly used comprehensive measurement and evaluation methods for urban development at home and abroad include the analytical hierarchy process (AHP), fuzzy comprehensive evaluation (FCE), factor analysis, entropy weight method (EW), grey relational analysis (GRA), and approximate ideal solution methods such as the ranking method (Topsis) [34–37]. Among them, the analytic hierarchy process is a simple method for making decisions on complex and fuzzy issues. Its core goal is to express, process, and evaluate the subjective judgement of complex issues in a concise and practical quantitative form. Although this method is highly systematic and practical, the importance of comparison and matrix judgement in this method is determined based on individual subjective scoring, which cannot overcome the defect of greater subjective arbitrariness [38]. EW is an evaluation method that draws on the idea of information entropy and comprehensively uses the entropy value of each indicator to make decisions. Its core goal is to determine the weight based on the degree of discreteness of the data itself and then to conduct a comprehensive evaluation. Although this method fully mines the information contained in the original data and can make the evaluation results more objective, because it only considers the information characteristics of the data itself and these information characteristics are affected by the quantity and quality of the data, sometimes the obtained weights may not match or may even contradict the actual importance. Factor analysis can synthesize important information on variables. However, the method for setting the number of factors and the weight of the synthetic factors needs to be preset, which is subjective. Therefore, in response to the above shortcomings, this paper determines the number of factors by calculating the cumulative variance contribution rate of factors and comprehensively screening out common factors according to two rules: a contribution rate not less than 85% and an eigenvalue greater than 1. For synthetic factor weights, the variance contribution rate ratio is often used, which has the disadvantage of subjectivity. The basic logic of the entropy weight Topsis method is to give weight to the basic data and judge the weight given based on the amount of information in the sample data to make the division between the sample data more significant; then, a comprehensive evaluation of the closeness of the evaluation factors of each indicator is conducted. The closeness of the distance between the evaluation sample and the ideal solution can be regarded as the straight-line distance between points, which improves the shortcomings of the factor analysis method by using the indirect distance to calculate the total factor score, thereby effectively combining the factor analysis and entropy weight Topsis methods. The advantages of this method is the evaluation results are more objective and scientific. Therefore, this paper uses an improved factor analysis method to evaluate the health of Chongzuo city and Nanning city in Guangxi. The specific steps are as follows:

Assuming n evaluation samples, each sample is evaluated with m indicators; then, its sample data are calculated as follows:

$$X=[\begin{array}{ccc} x_{11} & \cdots & x_{1m}\\ \vdots & \ddots & \vdots \\ x_{n1} & \cdots & x_{nm} \end{array}]$$ (1)

The original data indicators are averaged. The average value of each indicator is:

$${\bar{x}}_{j}=\frac{1}{n}\sum_{i=1}^{n}x_{ij}(j=1,2,\ldots ,m)$$ (2)

The original data are divided by the corresponding indicator mean to obtain the averaged data z_ij_:

$$z_{ij}=\frac{x_{ij}}{{\bar{x}}_{j}}(i=1,2,\ldots ,n;j=1,2,\ldots ,m)$$ (3)

The mean data covariance matrix C, whose matrix elements *c*_*ij*_(*i*,*j* = 1,2,…,*m*) are:

$$c_{ij}=\frac{1}{n-1}\sum_{k=1}^{n}{(z}_{ki}-{\bar{z}}_{i})(z_{kj}-{\bar{z}}_{j})$$ (4)

The correlation coefficient matrix R of the averaged data is calculated. The matrix element *r*_*ij*_(*i*,*j* = 1,2,…,*m*) is:

$$r_{ij}=\frac{u_{ij}}{\sqrt{u_{ii}}\sqrt{u_{jj}}}$$ (5)

The eigenvalues and eigenvectors of each correlation coefficient matrix R are calculated using the characteristic equation |*R*−*λI*| = 0. Find m nonnegative real roots and arrange them in descending order: *λ*_1_≥*λ*_2_≥⋯≥*λ*_*m*_≥0. *λ*_*i*_ is added into the equation system (*R*−*λI*)*α*_*i*_ = 0(*i* = 1,2,…,*m*) to find the corresponding eigenvector for *α*_1_, *α*_2_,…,*α*_*m*_, *λ*_*i*_, which is the variance of the i-th principal component y_i_ and reflects the magnitude of the role played by y_i_ in the evaluation.

The variance contribution rate *ω*_*k*_ and cumulative variance contribution rate *s*_*k*_ are calculated, and the variance contribution rate of the k-th principal component of the principal component is selected:

$$\omega_{k}=\frac{\lambda_{k}}{\sum_{i=1}^{m}\lambda_{i}}$$ (6)

For the first k principal components *y*_1_, *y*_2_,…,*y*_*k*_, the cumulative variance contribution rate is:

$$s_{k}=\frac{\sum_{j=1}^{k}\lambda_{j}}{\sum_{i=1}^{m}\lambda_{i}}$$ (7)

*ω*_*k*_ represents the amount of original information extracted from the k-th principal component, and *s*_*k*_ represents the amount of original information contained in the first k principal components extracted.

Assuming that the cumulative contribution rate of the first k principal components is greater than 85% and that their characteristic roots are all greater than 1, their characteristic roots are *λ*_1_, *λ*_2_,…,*λ*_*k*_; then, the weight is:

$$W_{i}=\frac{\lambda_{i}}{\sum_{i=1}^{k}\lambda_{j}}\left(i=1,2,\ldots ,k\right)$$ (8)

The comprehensive evaluation using the entropy-weighted Topsis method to synthesize the healthy city index (HCI) is:

$$x_{ij}=\left\{ \begin{array}{c} \frac{x_{ij}-min\{x_{1j,}x_{2j,\ldots ,}x_{Mj}\}}{\max\left\{x_{1j,}x_{2j,\ldots ,}x_{Mj}\right\}-min\{x_{1j,}x_{2j,\ldots ,}x_{Mj}\}},if\;j=\mathrm{benefit}\;\mathrm{attribute}\\ \frac{\max\left\{x_{1j,}x_{2j,\ldots ,}x_{Mj}\right\}-x_{ij}}{\max\left\{x_{1j,}x_{2j,\ldots ,}x_{Mj}\right\}-min\{x_{1j,}x_{2j,\ldots ,}x_{Mj}\}},if\;j=\mathrm{cost}\;\mathrm{attribute} \end{array}\right.$$ (9)

First, the indicators are standardized, and then the positive and negative ideal solutions are determined by calculating the weights and differentiation coefficients. Finally, the comprehensive factor, the healthy city index, is synthesized.

In summary, the difference between the improved factor analysis method and the traditional factor analysis method in this article is:

1. Differences in factor determination methods: Traditional factor analysis usually only relies on feature roots or variance contribution rates to determine the number of factors, which may be affected by factors such as data characteristics and sample size, resulting in inaccurate factor quantities. The method of combining feature roots and variance contribution rate adopted in this article can comprehensively consider the overall characteristics of the data and improve the accuracy of factor determination.
2. Differences in weight determination methods: Traditional factor analysis usually uses equal or subjective weighting methods to weigh each factor after obtaining factor scores. This method may be influenced by human factors, leading to bias in the evaluation results. The entropy weight Topsis method we use can determine the weights of each indicator based on objective information of the data, avoiding subjective weighting errors and making the evaluation results more objective and accurate.

The advantages of the improved factor analysis method proposed in this article compared to traditional methods are:

1. The accuracy of determining the number of factors: By combining feature roots and variance contribution rate to determine the number of factors, our method can more accurately identify the main factors affecting urban health status. Compared with traditional methods that only rely on feature roots or variance contribution, this method can comprehensively consider the overall characteristics of the data, avoid too many or too few factors, and thus improve the accuracy and reliability of the analysis results.
2. Objectivity of comprehensive evaluation: By using the entropy weight Topsis method to weigh the HCI (City Health Index), we can more objectively evaluate the urban health status of each city. The entropy weight method determines the weight of each indicator by calculating its degree of variation, avoiding bias and errors in subjective weighting. The Topsis rule is a method of evaluating the quality of objects based on the distance between ideal solutions and negative ideal solutions. It can comprehensively consider the relative relationship between various indicators and obtain more objective and accurate evaluation results.

**Fig 1:**
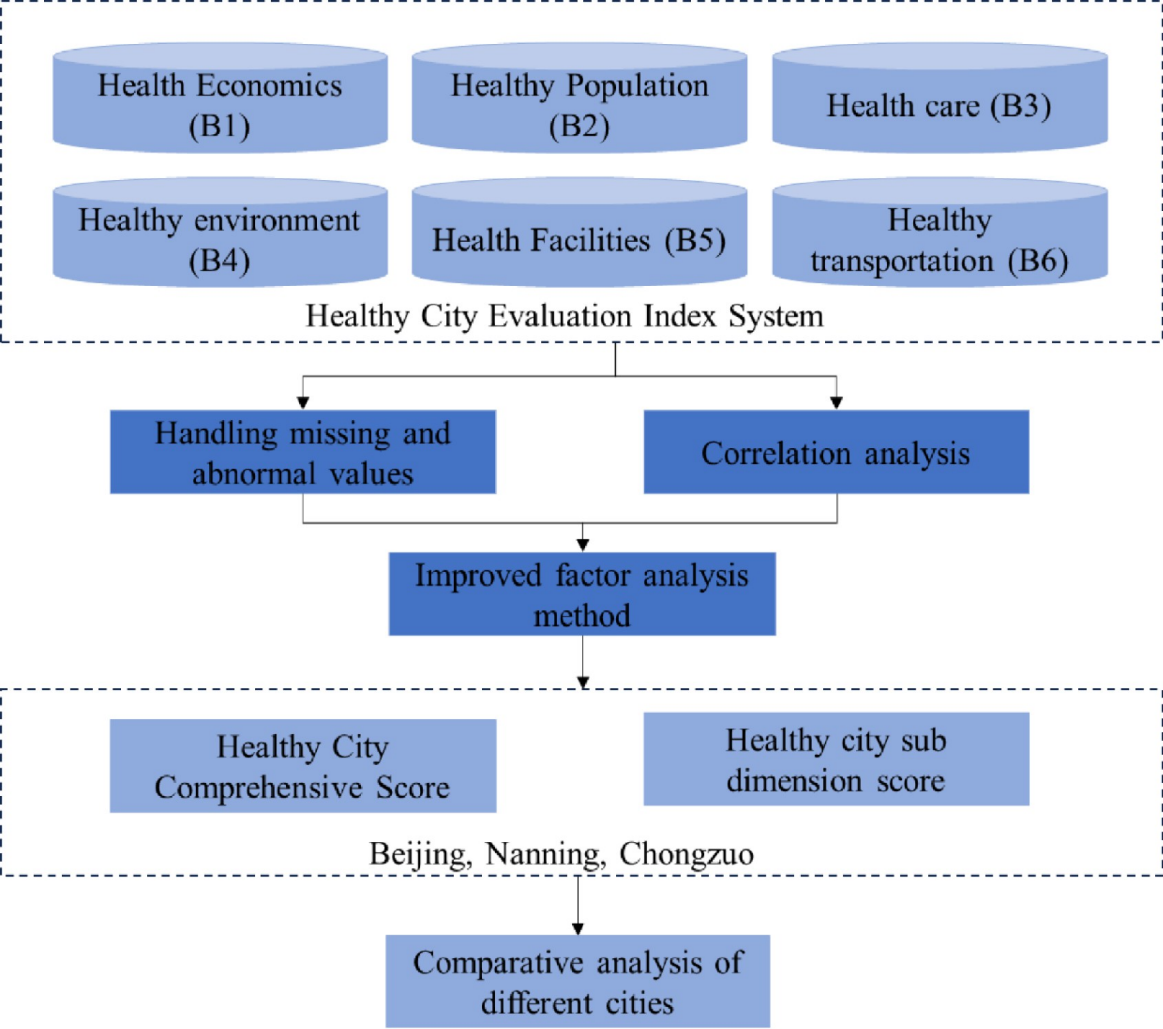
Research framework.

In addition, the research framework of this article is as Fig 1:

## 4. Empirical analysis

### 4.1 Data and establishment of the healthy city evaluation index system

This study adopts an improved factor analysis method for the comprehensive evaluation of healthy cities. First, the construction of the evaluation index system for healthy cities in the Guangxi Zhuang Autonomous Region is based on the "Healthy China 2030" Plan Outline, the "National Healthy City Evaluation Index System", and the action plans for healthy cities in various cities, combined with relevant literature [39, 40] and the availability and comprehensiveness of data from the Guangxi Zhuang Autonomous Region. We constructed an evaluation index system for healthy cities in the Guangxi Zhuang Autonomous Region. The index system includes six aspects, namely, a healthy economy (B1), healthy population (B2), healthy care (B3), healthy environment (B4), healthy facilities (B5), and healthy transportation (B6), and 26 secondary indicators were also selected. Then, based on the data of each indicator in the evaluation system, factor analysis was used to comprehensively evaluate cities such as Nanning and Chongzuo in Guangxi. The data used in this study covers the period from 2005 to 2022, aiming to comprehensively analyze the urban health status of each city during this period. All data are sourced from the National Statistical Yearbook, the official website of the National Bureau of Statistics of the People’s Republic of China, and the Statistical Yearbook of the Guangxi Zhuang Autonomous Region. The specific indicator system is shown in Table 1.

**Table 1:** Healthy city evaluation index system.

Indicator classification	Indicator name	symbol	unit
Healthy Economy (B1)	GDP	X1	Ten thousand yuan
	GDP per capita	X2	Ten thousand yuan
	GDP growth rate	X3	%
	Revenue	X4	Ten thousand yuan
	Industrial added value	X5	Ten thousand yuan
Healthy Population (B2)	Birth rate	X6	%
	Population death rate	X7	%
	Degree of urbanization	X8	%
Health care (B3)	Hospital Distribution	X9	place/person
	Distribution of registered nurses	X10	person/person
Healthy environment (B4)	Forest cover rate	X11	%
	Afforestation area	X12	hectare
	City Park green space area	X13	hectare
	Urban garden green space area	X14	hectare
	Municipal solid waste removal volume	X15	tons/day
	Urban sewage treatment rate	X16	%
	Total surface water supply	X17	10,000 tons
Healthy Facilities (B5)	Urban gas penetration rate	X18	%
	Urban drainage pipe length	X19	kilometre
	Urban water penetration rate	X20	%
	Urban construction land area	X21	square kilometres
	Number of public toilets in the city	X22	Place
	Urban natural gas supply	X23	10,000 cubic metres
Healthy transportation (B6)	Traffic density	X24	Ten thousand
	Proportion of postal business passenger transport	X25	%
	Railway passenger transport share	X26	%

For the construction of the indicator system, we first considered the availability of urban data in the Guangxi Zhuang Autonomous Region and the specific conditions of the cities in the Guangxi Zhuang Autonomous Region. Second, this indicator system covers all aspects of a healthy economy; that is, it reflects the systemic nature of the economy, including the GDP, GDP per capita, and GDP growth rate. At the same time, it also considers various other indicators, such as a healthy population, healthy medical care, a healthy environment, healthy facilities, and healthy transportation, which can more comprehensively reflect the health status of the city. Third, this indicator system meets systematic standards. Each indicator is not isolated but interrelated and influences each other. They can systematically reflect a city’s economic, social, environmental, and other health conditions. This indicator system not only includes static indicators that reflect the current situation of the city, such as the GDP and GDP per capita, but also includes dynamic indicators that reflect the trend in urban development, such as the GDP growth rate. This helps to understand the trends and patterns of healthy urban development in the Guangxi Zhuang Autonomous Region. In short, this method can be used to comprehensively evaluate the health of cities in the Guangxi Zhuang Autonomous Region.

### 4.2 Descriptive statistics and data distribution

**Fig 2:**
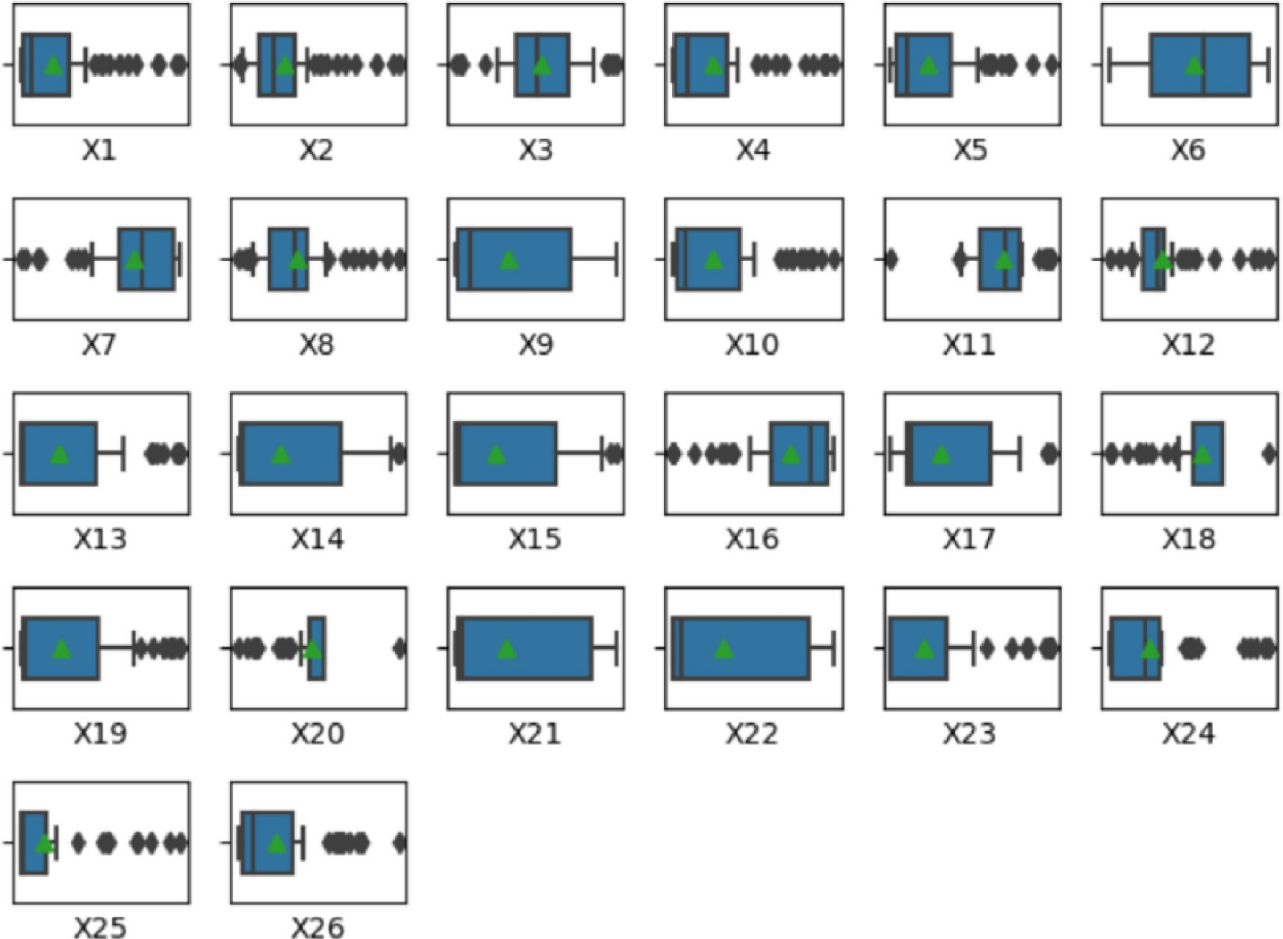
Data distribution of the healthy city indicator system.

The distribution of each indicator is displayed in the form of a boxplot, as shown in Fig 2:

From the box plot, we can see that there are significant differences in the distribution of each indicator, which shows that there are large differences in the development levels between cities. By observing the distribution of the boxes and outliers in the boxplot, we can see that each indicator has some larger values ​​and some smaller values, which indicates that there are some higher and lower levels of development between each city. Among them, it can be seen that the GDP growth rate (X3), birth rate (X6), afforestation area (X12), and urban drainage pipeline length (X19) show significant changes among different cities and years. The fluctuation of the GDP growth rate reflects the differences in economic development speed and strategies among cities. Beijing may place more emphasis on the quality and sustainability of economic growth, while Chongzuo and Nanning may place more emphasis on speed and scale. The relatively stable birth rate indicates that these cities have achieved good results in population policies, providing a stable population foundation for their sustainable development. The significant increase in afforestation areas, especially in certain cities and years, demonstrates the importance placed on ecological environment construction, which helps to improve urban ecological quality and address climate change. The increase in the length of urban drainage pipelines reflects the continuous improvement of urban infrastructure construction, which helps to enhance the flood control and drainage capacity of cities and ensure the safety of residents’ lives and property. The distribution characteristics of these indicator data and the reasons behind them provide us with a strong basis for a deep understanding of urban health status and improvement measures, further promoting the healthy development of cities.

### 4.3 Correlation analysis

**Fig 3:**
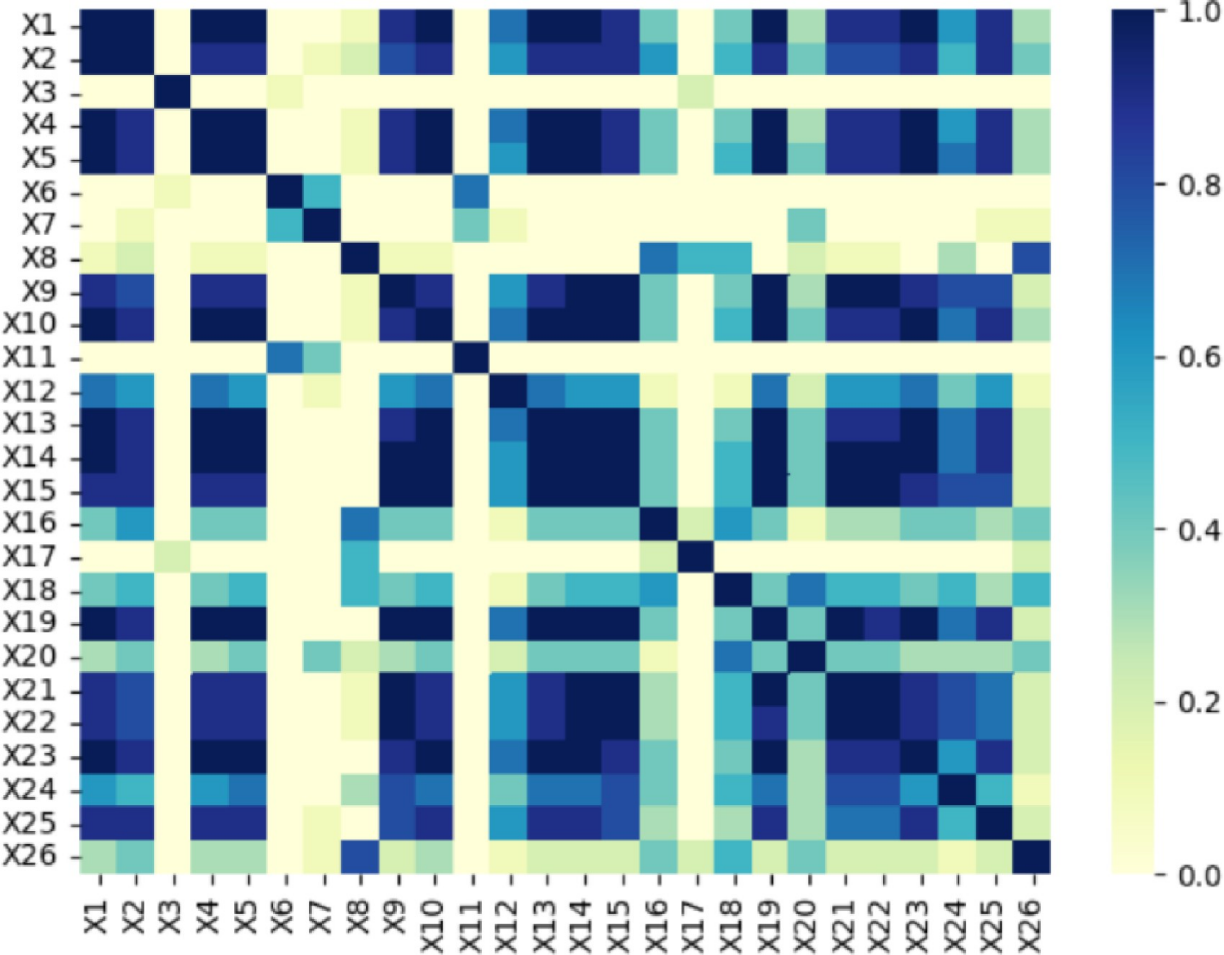
Correlation analysis.

It can be seen from the heatmap in Fig 3, In terms of the Healthy Economy (B1), there is a strong positive correlation between GDP (X1), GDP per capita (X2), revenue (X4), and industrial added value (X5) indicators, with correlation coefficients mostly exceeding 0.8, indicating a high degree of synergy among these economic indicators in measuring urban economic development. In terms of Healthy Population (B2), there is a certain correlation between birth rate (X6) and population death rate (X7), which reflects the dynamic changes in the natural population growth rate. However, compared to health economic indicators, its correlation is weaker due to more complex factors affecting population changes.

In terms of health and Health Care (B3), Health Environment (B4), Health Facilities (B5), and Health Transportation (B6), it can be seen that there is also a strong correlation between indicators within each category. For example, in healthcare, there may be a certain positive correlation between hospital distribution (X9) and registered nurse distribution (X10), as more hospitals may require more nurses to provide services. Similarly, in a healthy environment, indicators such as forest cover rate (X11) and afforestation area (X12) may also have a positive correlation with city park green space area (X13) and urban garden green space area (X14), as these indicators collectively reflect the situation of urban greening and ecological environment construction.

In summary, there is a high degree of correlation between the six types of indicators selected in this paper, and the value is essentially greater than 0.6. This high correlation shows that there is a close connection between these indicators and illustrates that the data we selected are scientific and comprehensive. These highly correlated indicators can reflect a common factor or multiple related factors, which increases the feasibility of the subsequent factor analysis. Factor analysis can be used to reduce the dimensionality of these six highly correlated indicators, thereby simplifying and clearly showing the connections and common characteristics of these indicators. These dimensionally reduced factors can more intuitively reflect the comprehensive performance of each city’s development level, allowing for a better comparison and analysis.

### 4.4 Evaluation results and discussion

#### 4.4.1 KMO test

However, before performing the factor analysis, the correlation between the original variables was first determined. When the correlation between variables is high, factor analysis can be used. The KMO test is generally used for judgement. It is generally believed that when the KMO value is greater than 0.5, factor analysis is possible. Table 2 shows the results of the KMO test.

**Table 2:** KMO and Bartlett’s test.

Kaiser‒Meyer‒Olkin Measure of Sampling Adequacy		0.837
Bartlett’s Test of Sphericity	Approx. Chi-Square	3654.683
	df	325
	Sig.	0.000

It can be seen from the test results that the KMO value is 0.837, which is greater than 0.80, reaching the judgement standard for performing a subsequent factor analysis. The significance is 0.00, which is less than 0.05, indicating that it is highly significant. Therefore, the data in this paper are suitable for factor analysis.

#### 4.4.2 Determination of the number of factors

The following shows the subsequent factor extraction and solution of the factor loading matrix. By calculating the cumulative variance ratio of each factor and requiring the eigenvalue to be greater than 1 as a judgement condition to select the number of factors, this paper finally extracted the top 6 main factors among the 28 indicators, corresponding to the 6 dimensions of a healthy city. In addition, the scree plot was used to determine the number of factors to select.

**Fig 4:**
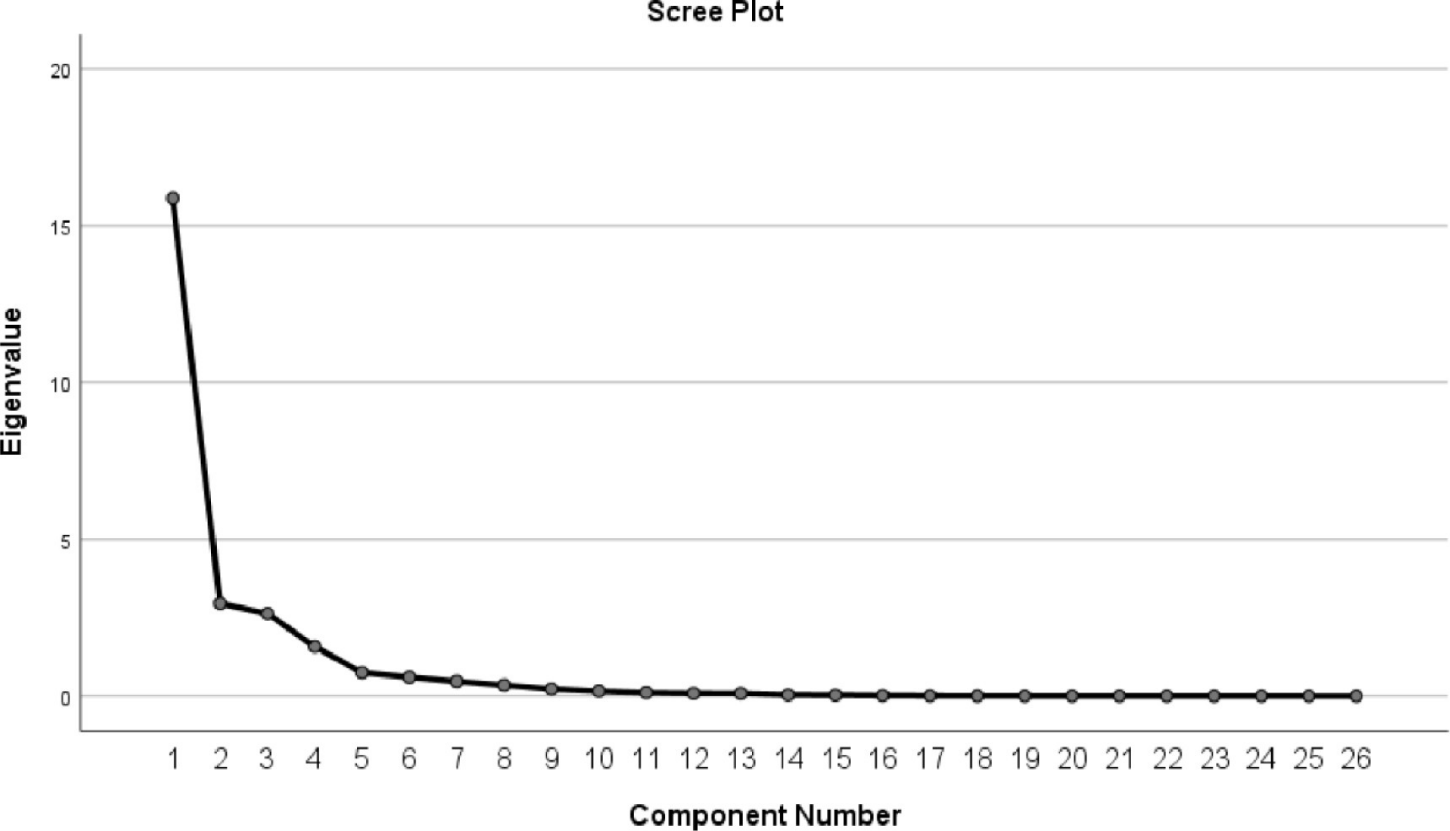
Scree plot.

Fig 4 also shows that when the number of factors is 6, the curve tends to stabilize. Therefore, it is most appropriate to select 6 factors. The total explained variance obtained by selecting 6 factors is shown in Table 3:

**Table 3:** Total variance explained.

Variable	Total	% of Variance	Cumulative %	Total	% of Variance	Cumulative %
1	15.8753	61.0590	61.0590	13.39	51.49	51.49
2	2.9558	11.3685	72.4274	3.32	12.77	64.25
3	2.6339	10.1303	82.5577	2.60	10.01	74.26
4	1.5791	6.0733	88.6311	2.22	8.52	82.78
5	0.7482	2.8778	91.5089	2.11	8.13	90.91
6	0.6034	2.3207	93.8296	0.76	2.92	93.83
7	0.4701	1.8080	95.6376			
8	0.3499	1.3456	96.9832			
9	0.2286	0.8793	97.8625			
10	0.1594	0.6130	98.4755			
11	0.1104	0.4246	98.9001			
12	0.0867	0.3336	99.2337			
13	0.0829	0.3188	99.5525			
14	0.0358	0.1375	99.6900			
15	0.0326	0.1253	99.8153			
16	0.0184	0.0709	99.8863			
17	0.0097	0.0373	99.9236			
18	0.0054	0.0207	99.9443			
19	0.0047	0.0180	99.9623			
20	0.0034	0.0131	99.9754			
21	0.0030	0.0115	99.9868			
22	0.0020	0.0076	99.9944			
23	0.0008	0.0030	99.9974			
24	0.0004	0.0014	99.9987			
25	0.0002	0.0007	99.9994			
26	0.0001	0.0006	100.0000			

The cumulative contribution rate of the six extracted principal components is above 90%, indicating that the first six factors can explain 90% of the information in the original data. The common factor variance extracted in this paper is shown in Table 4:

**Table 4:** Communalities.

Variable	Initial	Extraction
GDP (X1)	1.000	0.986
GDP per capita (X2)	1.000	0.973
GDP growth rate (X3)	1.000	0.897
Financial revenue (X4)	1.000	0.986
Industrial added value (X5)	1.000	0.977
Birth rate (X6)	1.000	0.759
Population death rate (X7)	1.000	0.891
Degree of urbanization (X8)	1.000	0.975
Hospital distribution (X19)	1.000	0.972
Registered Nurse Distribution (X10)	1.000	0.985
Forest coverage (X11)	1.000	0.917
Afforestation area (X12)	1.000	0.731
City park green space area (X13)	1.000	0.996
Urban garden green space area (X14)	1.000	0.994
Municipal solid waste removal volume (X15)	1.000	0.982
Urban sewage treatment rate (X16)	1.000	0.898
Total surface water supply (X17)	1.000	0.925
Urban gas penetration rate (X18)	1.000	0.889
Urban drainage pipe length (X19)	1.000	0.996
Urban water penetration rate (X20)	1.000	0.908
Urban construction land area (X21)	1.000	0.986
Number of public toilets in the city (X22)	1.000	0.977
Urban natural gas supply (X23)	1.000	0.994
Traffic density (X24)	1.000	0.943
Postal service passenger transport ratio (X25)	1.000	0.900
Proportion of railway passenger transport (X26)	1.000	0.957

It can be seen that the extracted common factors explain a high proportion of the 26 indicators, most of which are above 0.9, indicating that the extracted 6 indicators explain most of the information in the original data. After extracting common factors, this paper extracted a total of six factors, namely, F1, F2, F3, F4, F5, and F6.

#### 4.4.3 Healthy city index by dimension

The maximum variance method was used for factor rotation of the six factors, which increases the loading of each factor after rotation. The rotated component matrix is shown in Table 5.

**Table 5:** Component score coefficient matrix.

Variable	F1	F2	F3	F4	F5	F6
GDP (X1)	0.125	-0.114	-0.064	0.0324	-0.034	-0.086
GDP per capita (X2)	0.1136	-0.164	-0.049	-0.006	0.1017	-0.083
GDP growth rate (X3)	0.0135	0.1178	-0.042	-0.036	-0.244	-0.495
Financial revenue (X4)	0.1101	-0.085	-0.059	0.0137	-0.017	-0.021
Industrial added value (X5)	0.092	-0.042	-0.036	-0.007	0.007	-0.065
Birth rate (X6)	-0.024	-0.139	0.0962	-0.059	0.0152	0.2543
Population death rate (X7)	0.0006	-0.142	0.2766	0.108	-0.149	0.2284
Degree of urbanization (X8)	-0.095	0.1293	-0.074	0.3319	0.0768	0.3244
Hospital distribution (X19)	0.0399	0.1086	0.0002	-0.01	-0.042	-0.016
Registered Nurse Distribution (X10)	0.0835	-0.028	-0.019	-0.004	-0.005	-0.007
Forest coverage (X11)	0.0598	-0.382	0.0008	-0.173	0.2729	-0.078
Afforestation area (X12)	0.0401	0.0448	-0.062	0.1008	-0.213	0.6199
City park green space area (X13)	0.1002	-0.049	-0.019	-0.003	-0.034	-0.077
Urban garden green space area (X14)	0.065	0.027	0.0193	-0.012	-0.029	-0.031
Municipal solid waste removal volume (X15)	0.0491	0.0606	0.0361	-0.019	-0.024	-0.02
Urban sewage treatment rate (X16)	-0.017	-0.09	-0.097	-0.199	0.5951	-0.009
Total surface water supply (X17)	0.051	-0.058	-0.364	0.2272	-0.077	-0.048
Urban gas penetration rate (X18)	-0.056	0.0273	0.204	-0.034	0.3482	-0.427
Urban drainage pipe length (X19)	0.0864	-0.021	-0.007	-0.014	-0.024	-0.049
Urban water penetration rate (X20)	-0.064	0.0636	0.3852	0.1678	-0.038	-0.245
Urban construction land area (X21)	0.0037	0.1493	0.0768	-0.024	-0.013	0.0643
Number of public toilets in the city (X22)	0.001	0.1592	0.0765	-0.03	-0.012	0.0485
Urban natural gas supply (X23)	0.1341	-0.122	-0.053	-0.011	-0.037	-0.132
Traffic density (X24)	-0.153	0.3726	0.0779	-0.195	0.2703	0.588
Postal service passenger transport ratio (X25)	0.1605	-0.2	-0.085	-0.003	-0.032	-0.169
Proportion of railway passenger transport (X26)	0.0222	-0.021	0.0373	0.6458	-0.37	-0.149

By observing the score coefficients of each indicator on each factor, we can derive the main influencing factors of each factor. Among the F1 factors, the coefficients of GDP (X1) and GDP per capita (X2) were relatively high. These indicators have a greater weight on the F1 factor, indicating that they contribute more to the F1 factor. These indicators can be considered indicators of the economic development of a city. Therefore, the F1 factor reflects the healthy economy of a city. Among the F2 factors, the traffic density (X24) index had the largest weight, indicating that the F2 factor represents healthy transportation in a city. For the F3 factor, the weights of the population birth rate (X6) and the population death rate (X7) were relatively large, indicating that F3 represents a healthy population. For the F4 factor, the urban water penetration rate (X20) had the greatest impact, indicating that F4 represents a city’s healthy facilities. For the F5 factor, the urban sewage treatment rate (X16) and forest coverage rate (X11) had the largest weights, indicating that F5 represents a healthy environment in a city. Finally, the F6 factor represents the healthy medical care of a city. The summary results of the economic significance of these factors are shown in Table 6.

**Table 6:** Economic meaning of the factors.

Factor	Classification
F1	Healthy economy
F2	Healthy transportation
F3	Healthy population
F4	Healthy facilities
F5	Health environment
F6	Healthy care

**Fig 5:**
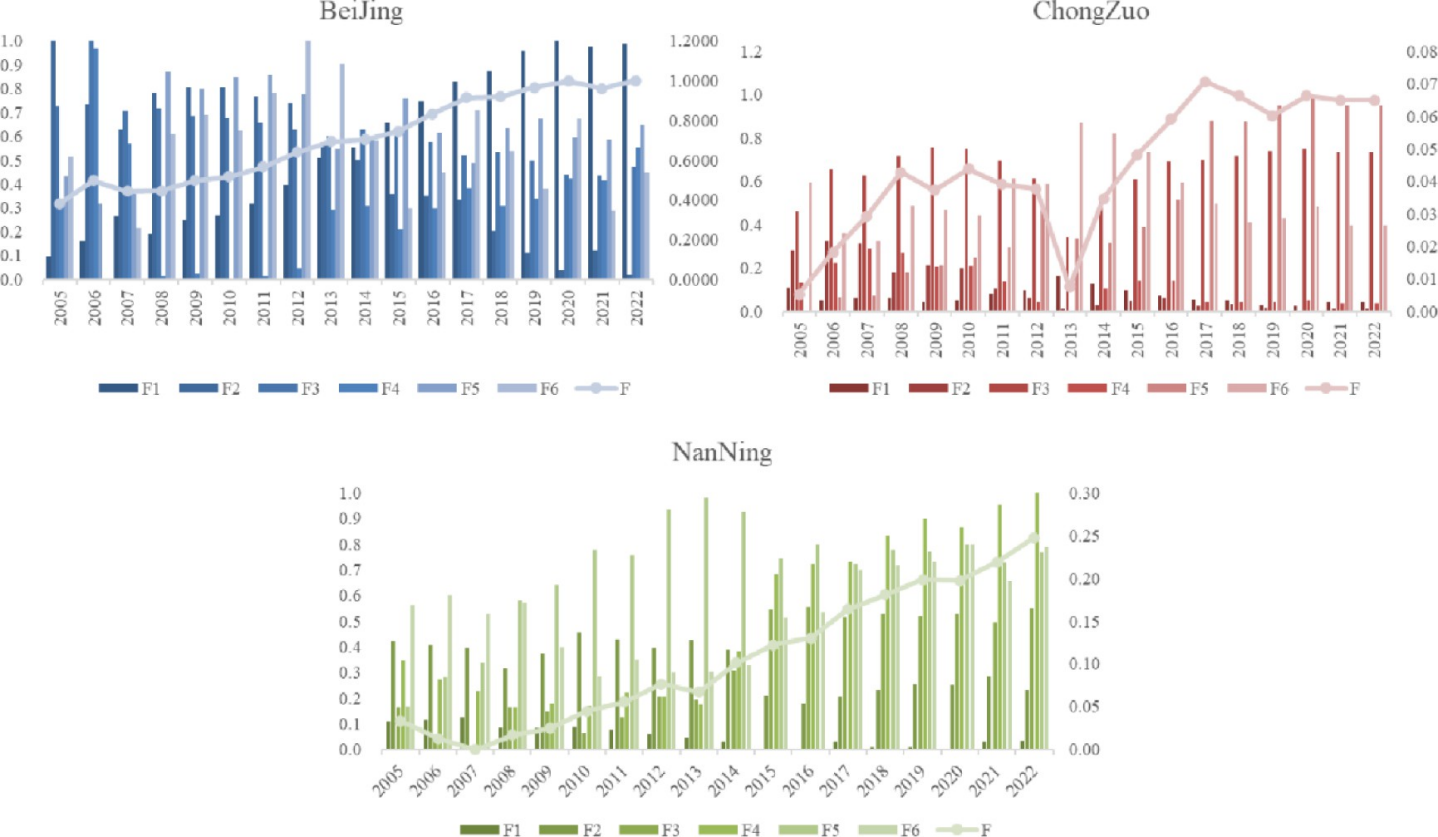
Growth trend of urban health evaluation.

The corresponding comprehensive factor score is the healthy city index (HCI). The entropy weight Topsis method was used to calculate the weighted weight, and the comprehensive factor F, the healthy city index (HCI), was synthesized. The comprehensive healthy city evaluation results of the indices and sub dimensional factor comparisons of Chongzuo city, Nanning city, and the benchmark Beijing are shown in Fig 5.

First, the healthy city index (F) reflects the explanatory power of all the factors in the original data. Therefore, the comparison of the healthy city index (HCI) evaluates the comprehensive performance of these cities in various aspects rather than being just a specific indicator. The healthy city index is also known as the healthy city index (HCI). The score of Chongzuo city shows that from 2005 to 2022, there is an overall upwards trend, indicating that the health level is developing and improving annually. However, its score decreased in 2013, mainly due to a decrease in the penetration rate of urban gas (X18) and urban water use (X20). However, the healthy city index of Chongzuo city subsequently showed a steady growth trend, indicating that the overall development of Chongzuo city improved. The healthy city index of Nanning steadily improved.

From a horizontal comparison perspective, we can see the differences in the healthy city index between different cities. These differences can be attributed to several factors, including the different economic strengths of the cities, the levels of social development, the environmental quality, and the transportation conditions. The higher the healthy city index is, the better the city’s performance in all aspects. Specifically, Beijing, with an HCI ranging from 0.2 to 1, shows strong performance in all aspects and serves as a national urban benchmark. Chongzuo and Nanning had lower HCIs, indicating areas for improvement. Overall, Nanning outperformed Chongzuo in terms of comprehensive development.

**Fig 6:**
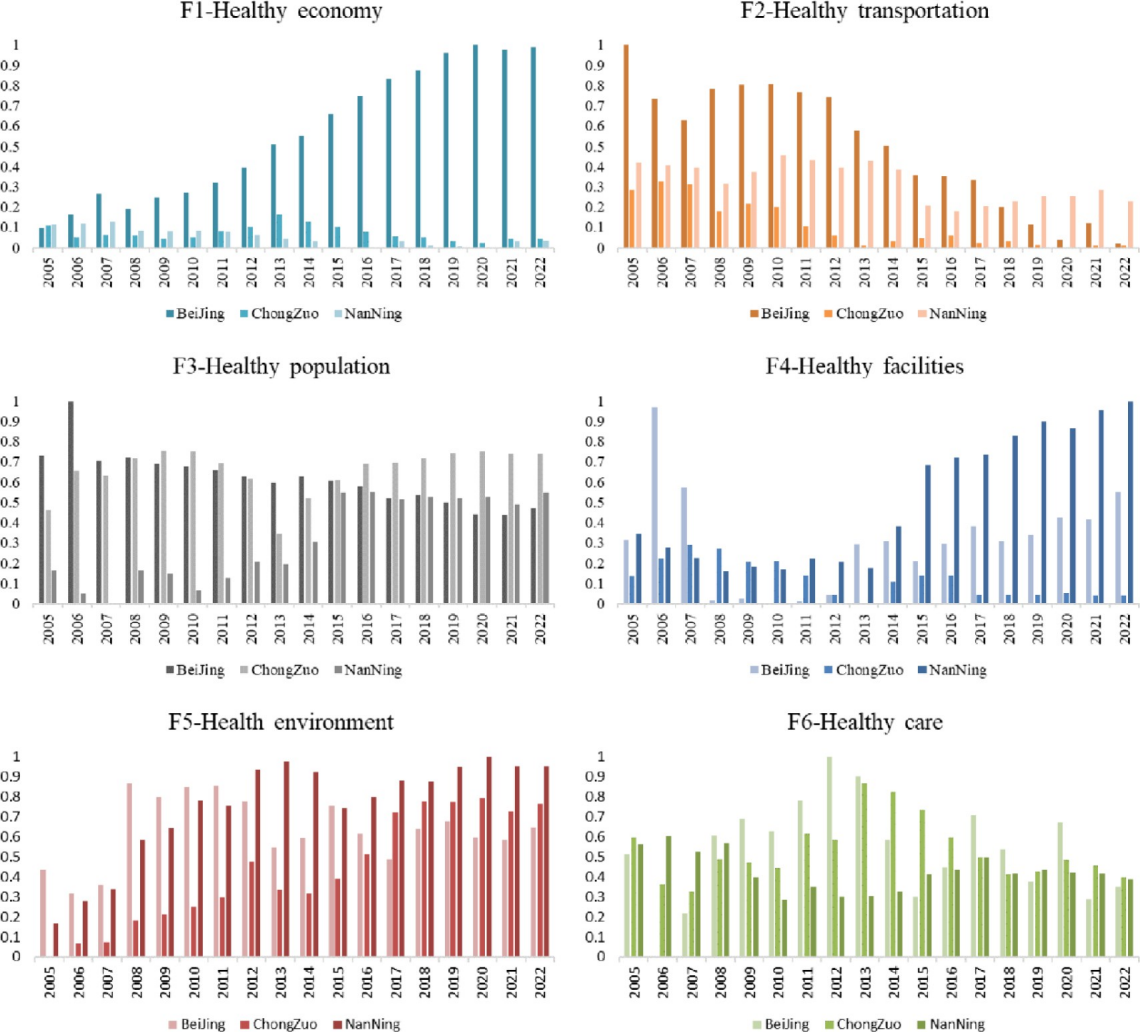
Comparison of the healthy city index scores for the six dimensions.

For a vertical comparison, we can observe the changes in the healthy city index of each city over different years. Overall, the healthy city index has generally increased over the years, reflecting positive urban development trends. A decreasing HCI may signal challenges. Fig 6 compares the three cities across the various dimensions.

**Fig 7:**
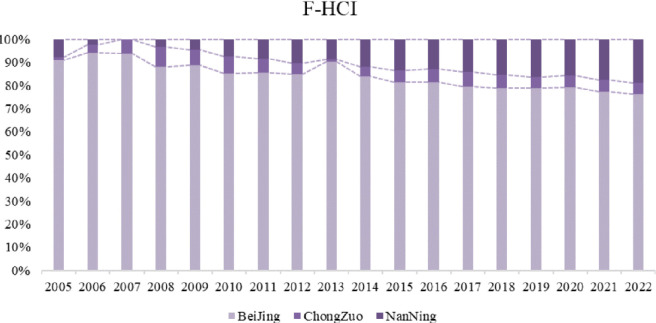
HCI changes.

First, we compare the healthy economy (F1) scores of these three cities horizontally every year, and Beijing is far ahead. From 2005 to 2011, Nanning’s healthy economy score was greater than that of Chongzuo, and after 2011, Chongzuo’s score was greater than that of Nanning’s. The reason may be that although the overall GDP of Chongzuo was lower than that of Nanning, the overall GDP growth rate of Chongzuo was higher than that of Nanning after 2011. In 2017, Chongzuo’s GDP grew by 9.3%, ranking second in Guangxi in terms of growth rate. In 2018, Chongzuo’s GDP grew by 11.3%, ranking first in Guangxi in terms of growth rate. In 2019, its growth rate of 8% is expected to continue to be among the highest in Guangxi. This means that although Chongzuo city may not be as good as Nanning city in terms of total output, its economic growth rate is faster, which greatly improves its healthy economy score. Second, in terms of healthy transportation (F2), before 2018, Beijing’s healthy transportation score was greater than that of Nanning and Chongzuo. However, after 2018, Nanning’s healthy transportation score was greater than that of Beijing due to its high traffic flow density. This may be because Nanning city is the capital of Guangxi Province, located in southern China, connecting the north and south, and Nanning has a superior geographical location, convenient transportation, and balanced passenger flow. In contrast, as the capital of China, Beijing has well-developed transportation facilities, but its high traffic density has led to more prominent traffic congestion problems, which affects the healthy transportation score. Third, in terms of the healthy population (F3) score, Chongzuo city has scored the highest in recent years. Chongzuo city has a higher healthy population score than Nanning and Beijing mainly due to its higher natural population growth rate. The natural population growth rate is an important indicator of the population growth of a city, and it involves the difference between the birth rate and the mortality rate. If the birth rate of a city is higher than the mortality rate, then the natural population growth rate of the city will be positive, indicating that the population is increasing. As a fifth-tier city, Chongzuo still has considerable development potential. Its population growth means more labour supply and potential consumer markets. An increase in the labour supply helps to enhance the economic development momentum of a city, providing more employment opportunities and creativity. Meanwhile, with population growth, consumer demand will also correspondingly increase, providing greater market space for urban economic growth. Fourth, in terms of the healthy facilities (F4) score, Nanning has had a faster growth rate than Beijing and Chongzuo since 2014. The urban water and gas penetration rates in Nanning are increasing, indicating that the facilities in Nanning are gradually improving and progressing. The Nanning Municipal Government has focused on urban construction and development strategies and is committed to creating a healthy and liveable city. In terms of urban infrastructure construction, the government has increased its investment, continuously improving urban gas and water supplies and drainage systems as indicated by increases in the urban gas penetration rate, urban drainage pipeline length, and urban water penetration rate. Fifth, for the healthy environment (F5) score, the healthy environment scores of Chongzuo city and Nanning city are relatively high, indicating that Nanning city has a greater degree of environmental protection, but there is still room for improvement, such as increasing the urban park and green space areas. This may be because Nanning is known as a "green city" with a high level of urban greening and numerous parks and green spaces, providing citizens with a healthy environment. In 2022, the rate of environmental air quality improvement in Nanning was 96.7%, with a PM2.5 concentration of 26 micrograms/cubic metre, ranking 16th among 168 key cities in China and 6th among provincial capital cities. From January to April 2023, the percentage of days with good air quality in the urban area was 99.2%, an increase of 4.2 percentage points year-on-year, and the "Nanning Blue" remained unchanged. Chongzuo city needs to make further efforts in terms of a healthy environment. Sixth, in terms of healthy medical care, Chongzuo city has scored higher in recent years due to a relatively balanced distribution of hospitals and nurses. This shows that the medical security of Chongzuo is relatively good. Even during the outbreak of novel coronavirus pneumonia in 2020, Chongzuo’s score was still slightly higher than that of Beijing. Chongzuo had no new infections for a long time. The hospital layout in Chongzuo city is relatively balanced, allowing patients to receive timely treatment when needed. In addition, medical personnel have strong execution abilities and can respond quickly, providing effective treatment for patients. In addition, the number of hospitals in Chongzuo city is moderate and balanced on a per capita basis. There are abundant Dendrobium officinale resources in Hualing, Nongfeng and other areas of Chongzuo city, Guangxi, which also provide good resources for research and the industrialization of traditional Chinese medicine. In addition to external support through policies, the Chongzuo Municipal Health Commission also issued a series of documents, such as the Example of Traditional Chinese Medicine Agreement in the Rehabilitation Period of Novel Coronavirus Infection, the Work Plan for Continuing Medical Education in 2022, and the "2022 Chinese Medicine Walk in China—Implementation Plan of Chinese Medicine Health Culture Theme Activity Project", which provided policy guarantees and implementation guidance for healthcare. The healthy city index trends for the six dimensions are shown in Fig 7.

**Fig 8:**
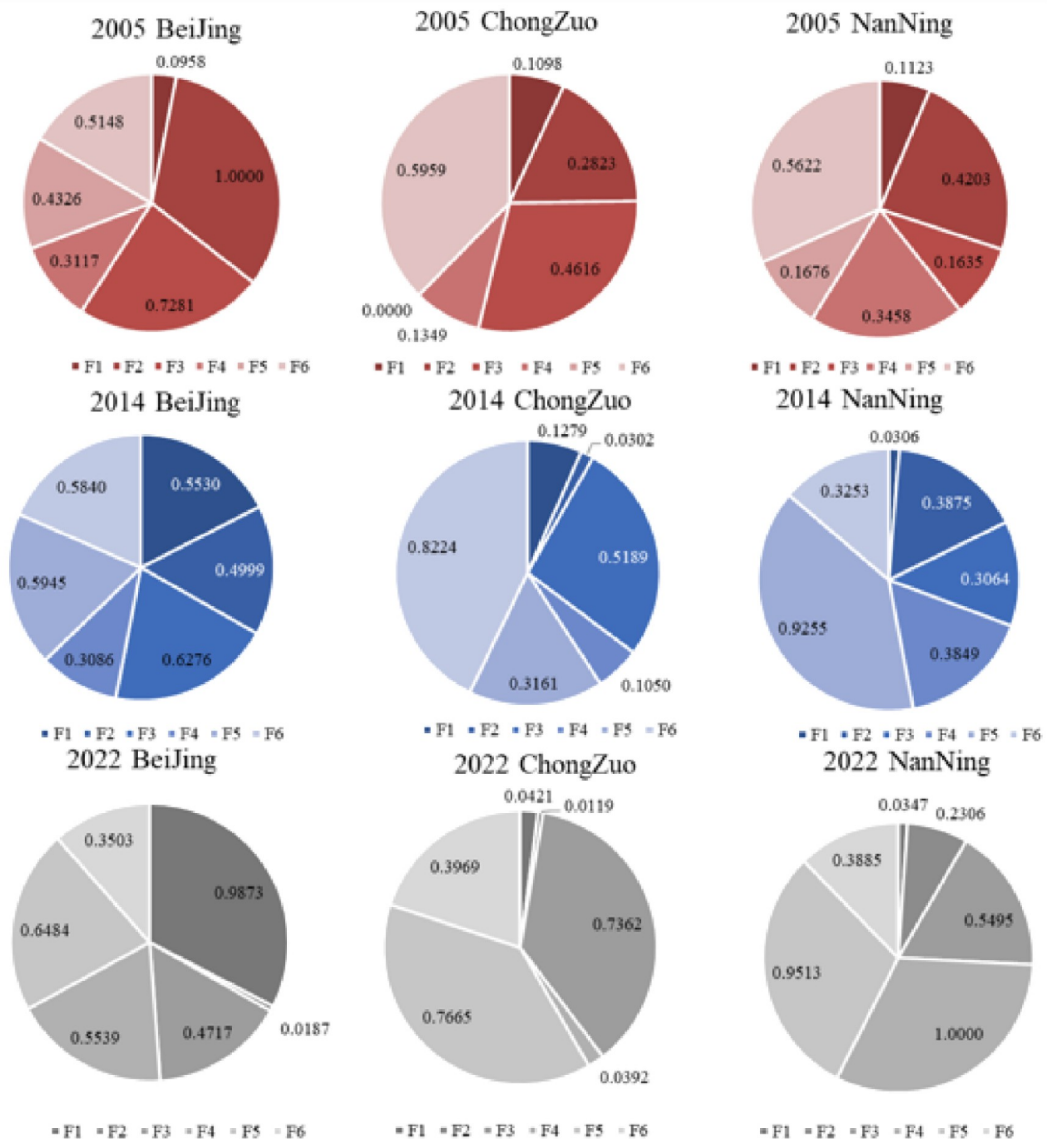
Comparison of the healthy city index scores of three cities in 2005, 2014, and 2022.

Overall, Beijing was stronger in all aspects and plays a leading role in China’s urban development. Nanning city, which is the capital of the Guangxi Zhuang Autonomous Region, contains most of Guangxi’s resources and has a relatively high level of health. Finally, for Chongzuo city, although its healthy city index is low, it scored high in the health and medical dimensions. Its advantages in medical management should continue to be leveraged, and its shortcomings in terms of economy and population should be compensated for. Next, we use 2005, 2014, and 2022 as examples to analyse and compare the scores of the three cities in Fig 8.

First, the healthy economy (F1) score of Chongzuo city is lower than that in the other dimensions. Higher scores were observed for the healthy population (F3), healthy environment (F5), and healthy care (F6) dimensions. The scores in these dimensions have increased annually, indicating that the population is gradually increasing, the environment is getting better, and healthcare is gradually improving. Particularly, in terms of a healthy environment (F5), the score has gradually increased to 0.7665 by 2022. To investigate the underlying reason, the Chongzuo Municipal Government launched the "Ecological Chongzuo" construction in 2005. This major action effectively promoted the improvement of a healthy environment in Chongzuo city. In 2014, the Chongzuo Municipal Government began revising the overall urban plan, focusing on urban greening, public facility construction and management, and enhancing the city’s image and quality. In addition, Chongzuo city was successfully established as a national healthy city in 2022. This event greatly promoted improvements in the urban hygienic environment. The healthy environment score of Nanning city also increased from 0.1676 points to 0.9513 points. The reason is that the Nanning Municipal Government has been committed to building a healthy environment. The government enhanced urban planning and environmental governance, improved public health facilities, promoted healthy lifestyles, and promoted the improvement of the urban health environment. In 2020, Nanning city was successfully established as a national healthy city. This major event greatly promoted the improvement of the urban sanitation environment. Economically, Nanning and Chongzuo have also shown a gradual upwards trend. However, they are still far below Beijing. The reason is that the economic development of Nanning and Chongzuo is still in its early stages, with a relatively simple industrial structure, mainly consisting of agriculture and manufacturing. As the capital of China, Beijing has more policy advantages and resource gathering capabilities, and its economic development is more diversified, as it includes high-tech industries, finance, service industries, among other sectors. In terms of healthy facilities (F4) and healthy transportation (F2), Chongzuo and Nanning have shown good development trends.

## 5. Conclusions

This paper selected data from 2005 to 2022 and established a healthy city evaluation index system. Twenty-six indicators from 6 dimensions were selected to evaluate the health status of Chongzuo city and Nanning city in the Guangxi Zhuang Autonomous Region, with Beijing serving as the benchmark city for comparison. An improved factor analysis method was used to synthesize the healthy city index. The research results indicated that the KMO value was 0.837, with a significance of 0.00, indicating high significance. Therefore, the data in this article were suitable for factor analysis. In addition, by utilizing factors with variance contribution rates greater than 85% and feature roots greater than 1, six factors were comprehensively determined and extracted as the optimal prime factors. These variables represent a healthy economy, healthy transportation, healthy population, healthy facilities, healthy environment, and healthy healthcare. By comparing the vertical and horizontal health indices of Chongzuo city, Nanning city, and Beijing city, we can draw the following conclusions: Chongzuo city has relatively high health and medical care scores, indicating that its medical security is relatively complete. This may be due to the relatively balanced layout of hospitals in Chongzuo city, which allows patients to receive timely treatment when needed. In addition, the number of hospitals in Chongzuo city is moderate and balanced with the local population. The healthy environment score of Nanning is relatively high, which may be related to the level of urban greening and the park green space area in Nanning. In addition, the Nanning Municipal Government has been committed to green transformation since the 18th National Congress, and the industry’s "green value" continues to rise. Nanning’s ecological civilization construction has become more solid, and the achievements have become more significant. Chongzuo city needs to make further efforts in the area of a healthy environment and needs to increase investment in environmental protection, such as by creating urban parks and green spaces. In terms of a healthy economy, Beijing is far ahead. This is because, as the capital of China, Beijing has relatively fast economic development and policy support. The healthy economy growth rate in Chongzuo city has been relatively fast, and the GDP growth rate has ranked among the highest in Guangxi in recent years. In terms of healthy transportation, Chongzuo city and Nanning city scored higher than Beijing city. This may be due to the high traffic density in Beijing causing traffic congestion. Chongzuo city and Nanning city are located in southern China and have convenient transportation and balanced passenger flow. Chongzuo city has the highest score for a healthy population, and a steadily growing population provides the city with stable human resources, which helps promote urban economic and social development.

## Supporting information

S1 Data (XLSX)
